# Computational Toxicology: Realizing the Promise of the Toxicity Testing in the 21st Century

**DOI:** 10.1289/ehp.1001925

**Published:** 2010-05-18

**Authors:** Ivan Rusyn, George P. Daston

**Affiliations:** 1 Department of Environmental Sciences and Engineering, School of Public Health, University of North Carolina–Chapel Hill, Chapel Hill, North Carolina, USA; 2 Miami Valley Laboratories, Procter & Gamble Company, Cincinnati, Ohio, USA

**Keywords:** computational toxicology, high-throughput screening, QSAR

## Abstract

**Background:**

The National Academies’ Standing Committee on Use of Emerging Science for Environmental Health Decisions held a meeting (21–22 September 2009 in Washington, DC) titled “Computational Toxicology: From Data to Analyses to Applications.” This commentary reflects on the presentations and roundtable discussions from the meeting that were designed to review the state of the art in the field and the practical applications of the new science and to provide focus to the field.

**Objectives:**

The meeting considered two topics: the emerging data streams amenable to computational modeling and data mining, and the emerging data analysis and modeling tools.

**Discussion:**

Computational toxicology is a subdiscipline of toxicology that aims to use the mathematical, statistical, modeling, and computer science tools to better understand the mechanisms through which a given chemical induces harm and, ultimately, to be able to predict adverse effects of the toxicants on human health and/or the environment. The participants stressed the importance of computational toxicology to the future of environmental health sciences and regulatory decisions in public health; however, many challenges remain to be addressed before the findings from high-throughput screens and *in silico* models may be considered sufficiently robust and informative.

**Conclusions:**

Many scientists, regulators, and the general public believe that new and better ways to assess human toxicity are now needed, and technological breakthroughs are empowering the field of toxicity assessment. Even though the application of computational toxicology to environmental health decisions requires additional efforts, the merger of the power of computers with biological information is poised to deliver new tools and knowledge.

Computational toxicology is the application of high-powered computing to manage and detect patterns and interactions in large biological and chemical data sets. Computational toxicology takes advantage of three significant technological breakthroughs: high-information-content data streams (e.g., from microarray or *in vitro* high-throughput screening experiments), novel biostatistical methods, and the computational power to analyze these data (Judson et al. 2009; Nigsch et al. 2009). Life scientists are acutely aware of the technologies that produce large data sets, but the steady increase in computational power is of equal importance in supporting discoveries at a systems level in understanding the interaction of environmental agents with biological systems, and how those interactions may produce adverse consequences. Perhaps because computer technology is so much a part of our daily lives, we have overlooked the fact that it is becoming a crucial element in the next great leap in our understanding of how exogenous agents affect living systems. In this commentary we reflect on the outcomes of the National Academies’ Standing Committee on Use of Emerging Science for Environmental Health Decisions meeting on “Computational Toxicology: From Data to Analyses to Applications,” sponsored by the National Institute of Environmental Health Sciences (National Academy of Sciences 2010). The overarching objectives of this meeting were to review the state of the art in computational toxicology and the practical applications of the new science and to provide focus to the field.

## Toxicology: A Science in Transition

Toxicology as a modern science has been active and productive in two complementary areas (Andersen and Krewski 2009). The first is the largely descriptive process of determining the effects of a large number of chemicals of commercial or environmental importance on the function of various organ systems, at different life stages, using animal models. This exercise has created a large knowledge base of the toxicologic effects of chemicals, usually at the organ or organismal level. Recent efforts to organize this type of information into a large, searchable database [Toxicity Reference Database (ToxRefDB); U.S. Environmental Protection Agency (EPA) 2010a] have yielded some interesting insights (Martin et al. 2009). The second area is the investigative process of identifying the mode of action of many of these agents, usually at the molecular or cellular level, which uses both *in vivo* and *in vitro* model systems (Harrill and Rusyn 2008). This latter path of investigation has been important in identifying a number of significant targets for toxicants. These two parallel tracks of research have been important in constructing a conceptual framework for toxicology that can be used to support risk assessment and public health decisions; however, these have a few significant drawbacks. One of the most important drawbacks is that both descriptive and mechanistic toxicology are labor and resource intensive and are too inefficient to comprehensively evaluate more than a fraction of the chemicals in commerce and the environment (Andersen and Krewski 2009). A proposed solution to this problem is to develop more rapid screening methods based on a mechanistic understanding of toxicity; however, mechanistic research has been reductionist in nature and may not be fully capable of characterizing the full spectrum of targets for agents that affect multiple systems at roughly the same concentration and/or have pleiotropic effects. There is a need, through a combination of high-information-content biology and computational modeling, to sew together the isolated threads of the reductionist mechanistic research into a framework that is predictive of the behavior of an intact biological system.

## Interdisciplinary Input into Toxicology

The field of computational toxicology is a synthesis of toxicology, biostatistics, systems biology, computer science, and many other relevant disciplines. Advances in computer-based approaches to modeling biological systems at different scales are becoming some of the key elements in facilitating the development of a predictive capacity for estimating outcomes or risk associated with exposure of organisms to drugs and environmental toxicants. Computational toxicology holds the key for effectively using high-dimensional data streams from computational chemistry, molecular biology, and systems biology. Importantly, the science of computational toxicology is reaching beyond basic research into the field of regulatory decision making and environmental health protection (Kavlock et al. 2009). The development of improved linkages across the source-to-outcome continuum—including the areas of chemical transformation and metabolism, better diagnostic/prognostic molecular markers, improved dose metrics, characterization of toxicity pathways, systems biology approaches, modeling frameworks, and uncertainty analysis—is a major objective of the science of computational toxicology. Equally important is its promise to provide improved predictive models for hazard identification, including the areas of quantitative structure–activity relationships (QSARs) and other computational approaches; improved pollution prevention strategies; and high-throughput screening of chemicals for safety. Finally, a multidisciplinary approach such as computational toxicology is required to address the uncertainties in quantitative risk assessment in dose–response assessment, cross-species extrapolation, and chemical mixtures, and to better understand the potential for chemicals to be human health and environmental hazards.

## New Data Sources: New Challenges and Opportunities

A number of data streams can be used to populate computational toxicology models (Judson et al. 2008). Toxicogenomics, proteomics, and metabolomics generate very large, information-rich data sets that are fodder for computational methods, although they each provide different information (Harrill and Rusyn 2008). These approaches provide a comprehensive assessment of gene expression, protein expression, or metabolite generation in a particular tissue, organ, or organism in response to a perturbation. This information has also been used as a way to identify pathways of response, at the molecular level, that are responsible for toxic outcomes.

High-throughput screening is another significant source of toxicologic information. In high-throughput screening, simple assays—such as receptor binding assays, enzymatic assays, or reporter gene assays—are conducted in multiwell-plate format in which hundreds or thousands of chemicals can be tested at once to query their effect on a single biological response (Houck and Kavlock 2008). This type of screening has been optimized by the pharmaceutical industry to evaluate extremely large combinatorial chemistry libraries to identify compounds with high activity for a specific molecular target. In toxicology, however, the application of the high-throughput screening is quite different: The ultimate outcome is lack of activity for key toxicity targets—a lack of activity that may ultimately be used as an indicator of lack of hazard or to assist in defining the mode of action. For example, the National Institutes of Health’s Chemical Genomics Center is conducting a large number of these high-throughput assays in parallel to investigate which biological processes are targets of environmental chemicals (Xia et al. 2008), as part of the ToxCast (Dix et al. 2007) and Tox21 (Collins et al. 2008) research programs. This effort involves screening > 1,000 chemicals, most of which also have a comprehensive *in vivo* data set, in several hundred *in vitro* assays that evaluate a specific aspect of toxicity. With all these data generated, computational modeling of biological systems can now rely on sufficient input details and begin *in silico* reconstruction of normal functions and prediction of major disease states. Using network models of biological systems, scientists may gain a better understanding of how cells sense their environment and respond to environmental stimuli. In turn, this understanding can help unravel complex relationships across biological systems and support a scientifically sound process of projecting human health risks posed by chemicals.

## “Chemical Toxicology”: Using Molecular Features for Predicting Toxicity

The prediction of toxicologic activity based on chemical structure (QSARs) was among the first applications of computation in toxicology. The first QSAR attempts were statistical in nature, based on the premise that toxicity could be correlated with certain molecular characteristics of the chemical agents that cause that particular kind of toxicity. These early models were limited in the number of parameters that could be modeled and tended not to be very predictive, especially for complex toxicities that can be produced through many different mechanisms of action, such as developmental toxicity or organ toxicity. Because these early models were not as successful or widely applicable as was hoped, the mainstream toxicology community is now only guardedly optimistic that QSAR models will be able to play a major role in prediction of chemical hazard. This is unfortunate, because there have been a number of advances in modeling chemical–biological interactions, based on a strong mechanistic understanding of toxicity, that are leading to improved structure–activity relationship (SAR) and QSAR models.

The most accessible and transparent approaches to prediction based on chemical structure have been expert rule-based SAR models, supported by large relational databases of toxicologic information that can be searched by chemical structure and substructure to identify analogs that can be used to make inferences about the toxicity of a new chemical (Richard et al. 2006). DSSTox (Distributed Structure-Searchable Toxicity; U.S. EPA 2010b) is one of these databases. It is a compilation of the toxicity data from a variety of sources in the peer-reviewed literature [e.g., TOXNET (National Library of Medicine 2010)] and gray literature [e.g., Toxic Substance Control Act Test Submission Database (TSCATS), and International Uniform Chemical Information Database (IUCLID5 2010)] literature that can be searched by chemical substructure. Expert-driven rules and best judgment from experts in medicinal chemistry can be used to identify the appropriate search strategies and analogs (Snyder et al. 2004; Wu et al. 2010). Although SAR still requires the involvement of experts, it demonstrates the value of chemical structure–based approaches in predicting toxicity. The next step is to incorporate the mechanistic understanding of experts into computational models, which is the realm of the next generation QSARs. These QSAR models are based on a solid framework of knowledge about the mechanisms that cause particular aspects of toxicity and are therefore likely to be highly predictive. For example, it is clear that a necessary chemical–biological interaction in chemical allergy is the covalent binding of the hapten to specific amino acid residues, and the relative potency of sensitizers is related to the speed and extent to which the binding takes place and this relationship can be modeled using QSAR (Gerberick et al. 2008).

Traditional QSAR models are developed based on chemical descriptors alone (Richard et al. 2006); however, the predictivity of most available toxicology-relevant QSAR models is quite limited, especially for *in vivo* toxicity end points (Zhu et al. 2008b). Historically, QSAR modeling in toxicology has been limited, in part, because of lack of mechanistically relevant biological data on hundreds of compounds. The recent availability of high-throughput and multidimensional (e.g., -omics) toxicity data on very large chemical libraries represents an intriguing avenue for further developments in computational toxicology via QSAR. Indeed, recent studies showed that the predictivity of QSAR models for *in vivo* toxicity is improved when *in vitro* testing results (i.e., biological descriptors) are combined with the traditional chemical descriptors (Zhu et al. 2008a).

## Applications of Computational Toxicology to Regulatory Decision Making

High-throughput cell-based and cell-free screening technologies (Xia et al. 2008) that can be applied to screen hundreds of chemicals in dozens of assays for the first time provide data that may be used for the toxicologic evaluation of their potential human health hazard, especially when combined with the knowledge of the chemical structure, metabolism, and disposition. The U.S. EPA and other organizations are viewing such screening programs as the first step to prioritize agents for targeted *in vivo* and/or *in vitro* testing (Judson et al. 2009). Although the prioritization process and hazard identification are important for the overall process of risk assessment, the *in vitro* data and computational predictions can be potentially useful in mode-of-action, dose–response, and exposure assessment and in understanding of the individual variability in the population.

The management, analysis, and interpretation of the new data now available for toxicologic assessment of safety require considerable computational resources but are likely to provide a great deal of insight into the possible mode of action of the chemicals under evaluation, as well as the value of the individual assays in supporting predictions for new chemicals. Even the analyses of the *in vivo* data sets that have been digitized from the historical paper records (Knudsen et al. 2009; Martin et al. 2009) have provided valuable insight into the strengths and weaknesses of the various animal models used to model potential human toxicity and into correlations between different manifestations of toxicity.

The modeling of complex data sets is helping to identify the key gene products that regulate biological activities that lead to toxicity. Many of the data for such modeling activities are from microarray experiments, along with proteomics and metabolomics. Toxicogenomics—the evaluation of all of the gene expression changes in a particular target tissue—has been particularly valuable in uncovering potential mechanisms of toxicity. Although some toxicants have highly specific targets, it is likely that most toxicants have the potential to interact weakly with a large number of targets, both complicating the process of defining how the toxicant produces its adverse effects and bringing into question whether all possible modes of action have been considered. Toxicogenomics addresses both questions. Analysis of these highly complex data sets is made possible through sophisticated computational methods that can elucidate concentration- and time-response patterns (Thomas et al. 2007) that are suggestive of effects on specific pathways (rather than just individual genes). This not only supports generation of hypotheses about mode of action but also provides knowledge for science-informed extrapolation for high to low dose and from animals to humans (Andersen et al. 2008).

Importantly, the dose-dependent transitions in modes of action for toxicant-mediated signaling, a phenomenon demonstrated for a number of toxicants (Naciff et al. 2005; Woods et al. 2007, 2009), call for the use of genomic, bioinformatic, and computational systems biology tools to understand different qualitative behaviors across dose levels. Dose–response studies uncovering molecular signatures of genes and pathways combined with the new computational systems biology tools for so-called developmental networks (Alon 2007) can provide data for mechanistic dose–response models that can integrate several areas in computational toxicology, infer low-dose behaviors, and assist in risk assessment as well as hazard identification.

New screening methods can also identify molecular targets and transcriptional regulators of key toxicity pathways that associate with *in vivo* end points, data that can be used as toxicity pathway–based biomarkers and as an input for predictive modeling of *in vivo* toxicity (Martin et al. 2010). Computational modeling can be used to evaluate how different gene products believed to be critical for a particular process interact. For example, critical genes in somite formation in the vertebrate embryo are being identified (McMahon et al. 2008) and can be modeled (Swat et al. 2009) to demonstrate the formation of somitic boundaries and positional information as somites are laid down on an anterior–posterior axis.

Physiologically based pharmacokinetic (PBPK) modeling is a methodology both for considering pharmacokinetic differences across species when estimating human risk from animal data and for evaluating the impact of pharmacokinetic variability on the dispersion of individual risks. Briefly, PBPK modeling attempts to describe the relationship between external measures of applied dose (e.g., amount administered or concentration in food, water, or air) and internal measures of delivered dose (e.g., amount metabolized or concentration in the tissue displaying the toxic response), using as realistic a description of mammalian physiology and biochemistry as is necessary and feasible (Clewell and Clewell 2008). PBPK modeling is an example of computational toxicology that has actually entered mainstream applications in risk assessment; it can address a critical gap in *in vitro*–to–*in vivo* extrapolations for relating results from improved *in vitro* models to real-world human exposure conditions and can assist in modeling dose–response behaviors in interactions of chemicals with biological systems.

Although cell-based *in vitro* models are being used extensively in toxicology studies as a means to evaluate the mechanisms of toxicity or to identify interactions of target compounds with metabolizing enzymes and transporters, the focus is frequently on either primary or immortalized cells of unknown genetic background. Importantly, the availability of the large bank of publicly available densely genotyped cells lines for lymphoblasts (e.g., Centre d’Etude du Polymorphisme Humain and Coriell Institute for Medical Research) and for cancer (e.g., NCI-60 panel; Developmental Therapeutics Program, National Cancer Institute, National Institutes of Health, Bethesda, MD) shows promise for *in vitro* screening that can appropriately consider the genetic variability in the population (Welsh et al. 2009). Although not without limitations, studies in genetically defined *in vitro* models may provide critical information for science-based considerations for both intra- and interspecies uncertainty factors used in risk assessment.

## Conclusions

The areas of promise for computational toxicology were discussed by the scientists from the government regulatory agencies, nongovernmental organizations, academia, and industry at a September 2009 meeting titled “Computational Toxicology: From Data to Analyses to Applications,” convened by the National Academies’ Standing Committee on Use of Emerging Science for Environmental Health Decisions and sponsored by the National Institute of Environmental Health Sciences (National Academy of Sciences 2010). Applications of computational approaches to predictive toxicology will be important in prioritizing chemicals for further testing and in uncovering mechanistic information that is valuable in tailoring testing programs for each chemical in an informed way, as well as supporting risk assessment. Computational methods also hold promise in other areas of the risk assessment process, particularly in estimating the extent of variability in response in the human population, in supporting more sophisticated aggregate exposure assessment, and in providing a pragmatic approach to evaluating the risks posed by cumulative exposure to mixtures of compounds.

It was clear that these are still early days for the application of computational toxicology to risk assessment and chemical regulation, but there are already examples where application of computational toxicology is taking place. For example, relational databases that can be searched by chemical substructure are being used to make predictions about the toxicity of new chemicals based on their similarity to chemicals for which the toxicity potential has been evaluated. The U.S. EPA has recently evaluated the use of toxicogenomics data to support its risk assessment approaches for phthalate esters (U.S. EPA/Office of Research and Development/National Center for Environmental Assessment 2009). Because the desire for computational approaches exceeds the pace at which practical applications are coming online, many of the regulatory agency participants at the meeting expressed frustration that their expectations were not being met. It will be important in the future to manage these expectations and to make sure that there are enough short-term applications so that the long-term research programs, which are necessary to meet the full potential of computational toxicology, can be supported.
